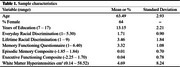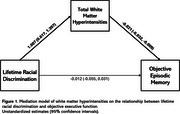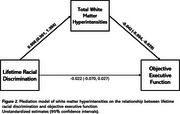# White Matter Hyperintensities Mediate the Effect of Racial Discrimination on Cognition

**DOI:** 10.1002/alz70860_106851

**Published:** 2025-12-23

**Authors:** Robrielle M. Pierce, Kiana A. Scambray, Monica E. Walters, Jordan D. Palms, Emily P. Morris, Ketlyne Sol, Lauren Taylor, Vivian Ku, Laura B. Zahodne

**Affiliations:** ^1^ University of Michigan, Ann Arbor, MI, USA

## Abstract

**Background:**

Racial discrimination is associated with poorer cognitive health, but underlying mechanisms are unclear. Greater discrimination is associated with more white matter hyperintensities (WMH), an indicator of cerebrovascular disease burden and important factor for cognition. WMH may serve as a link between discrimination and cognitive functioning via stress‐related inflammatory and vascular processes. This cross‐sectional study aimed to examine whether WMH mediated associations between racial discrimination and (1) subjective memory, (2) objective episodic memory, and (3) objective executive functioning in Black older adults.

**Method:**

Data were obtained from 351 non‐Latine Black participants aged 55+ without dementia from the Michigan Cognitive Aging Project. Discrimination attributed to race or skin color was operationalized with the Everyday Discrimination and Major Experiences of Lifetime Discrimination Scales. Subjective memory concerns were measured with the Memory Functioning Questionnaire. Episodic memory and executive functioning composite scores were created based on a comprehensive neuropsychological battery. Total WMH was quantified from T2 FLAIR images. RStudio statistical package PROCESS macro was used to examine six separate mediation models, controlling for age, gender, and education.

**Result:**

More lifetime, but not everyday, racial discrimination was associated with worse executive functioning (b=‐0.062; 95% CI=‐0.111, ‐0.012) and greater subjective memory concerns (b=0.119; 95% CI=0.045, 0.192), but not objective memory. More lifetime, but not everyday, racial discrimination was associated with greater WMH. Greater WMH was associated with worse objective memory and executive functioning, but not subjective memory. There were indirect effects of lifetime racial discrimination on objective memory (b=‐0.021; 95% CI=‐0.041, ‐0.010) and executive functioning (b=‐0.040; 95% CI=‐0.072, ‐0.022) through WMH, but not subjective memory. There was a direct effect of lifetime racial discrimination on subjective memory (b=0.103; 95% CI=0.027, 0.179), but not objective memory or executive functioning.

**Conclusion:**

These findings highlight the role of WMH as a potential mechanism linking racial discrimination to cognitive health. Results suggest that major life events of racial discrimination are particularly relevant to objective, rather than subjective, cognition, highlighting an important intervention target to improve cognitive health equity. More work is needed to identify policies targeting race‐based discrimination to reduce WMH burden and improve cognitive outcomes among older Black adults.